# Underutilized legumes: nutrient status and advanced breeding approaches for qualitative and quantitative enhancement

**DOI:** 10.3389/fnut.2023.1110750

**Published:** 2023-05-18

**Authors:** Ipsita Samal, Tanmaya Kumar Bhoi, M. Nikhil Raj, Prasanta Kumar Majhi, Sneha Murmu, Asit Kumar Pradhan, Dilip Kumar, Amit Umesh Paschapur, Dinesh Chandra Joshi, P. N. Guru

**Affiliations:** ^1^Department of Entomology, Faculty of Agriculture, Sri Sri University, Cuttack, Odisha, India; ^2^Forest Protection Division, ICFRE-Arid Forest Research Institute, Jodhpur, India; ^3^Division of Entomology, ICAR-Indian Agricultural Research Institute, New Delhi, India; ^4^Regional Research and Technology Transfer Station, Odisha University of Agriculture and Technology, Keonjhar, Odisha, India; ^5^ICAR-Indian Agricultural Statistics Research Institute, New Delhi, India; ^6^ICAR-National Rice Research Institute, Cuttack, Odisha, India; ^7^ICAR-National Institute of Agricultural Economics and Policy Research, New Delhi, India; ^8^ICAR-Vivekananda Parvatiya Krishi Anusandhan Sansthan, Uttarakhand, India; ^9^ICAR-Central Institute of Post-Harvest Engineering and Technology, Ludhiana, India

**Keywords:** underutilized legumes/orphan legumes, food and nutritional security, climate resilience, genetic improvement, comparative genomics

## Abstract

Underutilized/orphan legumes provide food and nutritional security to resource-poor rural populations during periods of drought and extreme hunger, thus, saving millions of lives. The Leguminaceae, which is the third largest flowering plant family, has approximately 650 genera and 20,000 species and are distributed globally. There are various protein-rich accessible and edible legumes, such as soybean, cowpea, and others; nevertheless, their consumption rate is far higher than production, owing to ever-increasing demand. The growing global urge to switch from an animal-based protein diet to a vegetarian-based protein diet has also accelerated their demand. In this context, underutilized legumes offer significant potential for food security, nutritional requirements, and agricultural development. Many of the known legumes like *Mucuna* spp., *Canavalia* spp., *Sesbania* spp., *Phaseolus* spp., and others are reported to contain comparable amounts of protein, essential amino acids, polyunsaturated fatty acids (PUFAs), dietary fiber, essential minerals and vitamins along with other bioactive compounds. Keeping this in mind, the current review focuses on the potential of discovering underutilized legumes as a source of food, feed and pharmaceutically valuable chemicals, in order to provide baseline data for addressing malnutrition-related problems and sustaining pulse needs across the globe. There is a scarcity of information about underutilized legumes and is restricted to specific geographical zones with local or traditional significance. Around 700 genera and 20,000 species remain for domestication, improvement, and mainstreaming. Significant efforts in research, breeding, and development are required to transform existing local landraces of carefully selected, promising crops into types with broad adaptability and economic viability. Different breeding efforts and the use of biotechnological methods such as micro-propagation, molecular markers research and genetic transformation for the development of underutilized crops are offered to popularize lesser-known legume crops and help farmers diversify their agricultural systems and boost their profitability.

## 1. Introduction

Food security has long been a vexing subject that is yet to be resolved. A significant increase in population and a decline in available water and arable land are limiting agricultural viability (1). Furthermore, global climate change is a huge impediment to existing production processes, while some arable land may become inaccessible for agriculture in the future as ice melts. The current global situation is so grave that millions of people are going hungry, and many are dying as a result of malnutrition. Since the green revolution began in the 1960s, greater irrigation and the use of inputs like chemical fertilizers and pesticides have enhanced the productivity of the world’s major staple crops (especially wheat, maize and rice) (2). However, future food security is threatened due to human dependence on less than 1% of edible plant species, as well as the detrimental effects of climate change and resource limitation (2, 3). Having constant, reliable, and low-cost access to a diverse range of healthy foods across a range of dietary preferences is what is meant by the term “food security” (4). Over the last decade, more than 800 million individuals have been reported as chronically undernourished globally, with around 821 million instances recorded in 2017 (5). With rapid population increase and quicker loss of non-renewable natural resources, it has become critical to diversify modern intensive agriculture to suit the diverse human demands. Diversifying agricultural output has several benefits for farmers and the local community, including biodiversity conservation, enhanced soil and plant health, less vulnerability to pests, diseases, and extreme weather events. Therefore, the present sustainable development goals (SDGs) emphasize agricultural diversification through the use of undervalued and forgotten crops.

After cereals, which have been a staple of the rationed human diet for millennia (6–8), legumes have been identified as the second most relevant plant source for human and animal dietetics (9), especially in drought and famine situations (10). In order to satisfy the ever-increasing need for vegetable proteins, there has been a recent shift in focus toward underused legumes as a potential source of blooming new alternative protein sources (11). Reduced malnutrition could help reduce global disease burden by 32%, as estimated by the World Health Organization (WHO): 115 million children under the age of five are stunted; 462 million adults are underweight; 41 million children are overweight and obese; and 1.9 billion adults are overweight and obese (12). Success in introducing such novel legumes to a region has the potential to increase dietary diversity and reduce the prevalence of malnutrition. A country’s agrarian standing may also be bolstered in this way, since it will provide new chances for domestic businesses, raise living standards for locals and decrease the need to rely on foreign suppliers. Many types of legumes, whether they are wild or domesticated, are now only farmed in a tiny fraction of their original range. Orphan legumes/underutilized plants like these have the potential to contribute to sustainable agriculture. Thanks to the fact that they can be bred for desirable traits like increased nutrition and resistance to stresses (13). Very little is known about the lesser utilized crops, and knowledge regarding these is limited to areas with special cultural or historical significance. Although they have become increasingly main-stream, they have received less international attention (14). The family of legumes, often known as Fabaceae or Leguminaceae, is the third largest in the world in terms of total population. The legumes are reported to be cultivated in broad variety of environments, from deserts to woodlands, alpine to aquatic and from the African rainforests to the Amazon. It is believed that they originated in Africa, made their way to South America and then spread north to North America and ultimately the rest of the world. Large populations of these may be found all throughout the South Asian continent and the Indo-Pacific. Caesalpinioideae, Mimosoideae and Papilionoideae are the three subfamilies based on floral characteristics, which make up this large family. The Papilionoideae, with 476 genera and over 14,000 species (15), is the most numerous, followed by the Mimosoideae with 77 genera and roughly 3,000 species and the Caesalpinioideae with 162 genera and roughly 3,000 species. However, only a small number of legumes including peas, soybeans and a few types of beans are widely consumed. There are still about 700 genera and 20,000 species that can be developed and incorporated into the mainstream for domestication and human consumption.

Increased visibility of underutilized legumes’ stems from the fact that, they can better endure to a range of climatic conditions (5). Since legumes are one of the most numerous plant families and contain a lot of proteins, we have many possibilities to pick from. It is unfortunate because legumes are an excellent substitute for those trying to eliminate or reduce their intake of animal products. Cowpeas, pigeon peas, and Bambara groundnuts are underrated legumes that can add a lot of nutritional value to our diets. Such legumes are widely produced in areas where they have traditionally functioned as a staple crop, but are consumed by significantly fewer people in other parts of the world. In addition to supporting the small farmers who grow them, eating more of these legumes will increase your intake of protein and other essential nutrients. Additionally, underutilized legumes can aid in the worldwide fight against hunger and malnutrition. People in these areas considerably benefit from these crops because of their resilience and hardiness, which are especially important given the scarcity of other food sources. Their increased resistance to climate change, pests, and diseases makes them a potentially game-changing crop for the second green revolution in farming. There are multiple ways in which the widespread adoption of underutilized legumes can contribute to the advent of a second green revolution. Underutilized legumes are a highly nourishing and sustainable food supply that can aid in increasing food security and decreasing poverty in many regions of the world. Further, these crops are a versatile and robust, that may be grown in a wide variety of agroecological and production systems and can improve soil health and increase food production. Further, increasing the use of underutilized legumes can boost economic growth and alleviate poverty in many regions of the world. The local economy can be stimulated and new jobs created by expanding the production and consumption of these legumes.

This article examines the potential of many obscure legume plants for future sustainable agricultural solutions to hunger and nutritional challenges, as well as genetic and molecular approaches promoting their wider usage and adaptability in modern agriculture.

## 2. Nutritional status and health benefits of underutilized legumes

One of the most urgent concerns of the time is ensuring everyone has enough to eat. The nutritional, agronomic, economic and ecological ramifications of relying so heavily on a small number of essential basic crops have limited global food security over the years (16). Stunted growth (especially in children under the age of 5) as well as child and infant mortality are largely attributable to malnutrition (17). Reduced economic growth and productivity are the result of both under nutrition (leading to stunted growth, underweight and mineral and vitamin deficiency) and over nutrition (leading to cancer, diabetes mellitus, stroke and heart disease) (12). The World Health Organization (WHO) reports that 462 million people are underweight, 115 million children under the age of five are stunted, 41 million youngsters are overweight or obese and 1.9 billion adults are overweight or obese. Disease rates worldwide may be lowered by 32% if malnutrition were reduced (12). Abnormal fetal brain physiology and morphology have been linked to malnutrition, particularly protein shortage (18). Death rates, medical expenses, and recovery times are all impacted by poor nutrition (19–22). Nutrient-rich but often overlooked legumes can improve both health and food security (23). They are low-cost and a good source of protein at a time when the consumption of animal products is prohibited due to potential health risks. Consequently, there has been an increase in the promotion and endorsement of plant-based protein as a means of satisfying the demands of the people (3). Beans, lentils, chickpeas, peas, and soybeans are currently some of the most widely farmed and consumed legumes. The Americas, Asia, and Africa are just some of the places where these legumes are cultivated. Numerous legume types exist that are underutilized since they are not as commonly grown or consumed as the big legumes. These legumes are not only an excellent food source, but also beneficial to soil health and erosion control. In conclusion, large legumes are notable for being an essential food crop and staple in many parts of the world. In addition to their potential to boost food security and soil health, underutilized legumes are a valuable resource in and of themselves. Bambara groundnut (*Vigna subterranean*), Jack bean (*Canavalia ensiformis*), Lima bean (*Phaseolus lunatus*), and sword bean (*Phaseolus edulis*) had their nutritional profiles analyzed by Soetan and Adeola (24). The nutritional needs of humans can be adequately met by consuming plants like *Cassia hirsuta* L., which has a high protein, lipid, potassium, fiber, carbohydrate, and energy content (24–26), and velvet bean (*Mucuna pruriens*), which has optimal crude protein, lipid, fiber, carbohydrate, energy, calcium, potassium, phosphorus, zinc, manganese, and magnesium content. Since different parts of underutilized legumes can be consumed, picky eaters can still get the same amount of nutrients. The blossom, seeds, pod case, immature pods, tuberous roots, and leaves of some underutilized legumes like winged bean, fenugreek, marama bean, and African yam bean are all edible. This confirms their wider usage over traditional legumes. The details of nutritional and anti-nutritional compounds in underutilized legumes have been furnished in Table 1.

**TABLE 1:** Nutritional and anti-nutritional compounds in Underutilized legumes.

SL. No.	Crop name	Protein content	Fat/oil content/fatty acid	Anti-nutritional compound								References
				**Phenolics**	**Trypsin inhibitor**	**Phytic acid**	**Oligosaccharide**	**Tannin**	**Phytate**	**HCN**	**Oxalate**	
1	*Tylosema esculentum*	34.71%	34.0 g/100 g of seed	24.6 mg/100 mg	NA	NA	NA	NA	NA	NA	NA	(146)
2	*Vigna subterranea*	15–25 %	5.2–6.4 %	NA	6.75–19.08 units/mg	46 mg/100 g	NA	NA	NA	NA	NA	(147)
3	*Canavalia spp*	300 g/kg	5.20%	NA	NA	NA	0.61–9.84 g/100 g	NA	NA	NA	NA	(148)
4	*Psophocarpus tetragonolobus*	34.3–40.7%	1.91%	NA	NA	NA	NA	1.69–2.57%	3.78–9.38 mg/100 g	NA	NA	(149)
5	*Parkia biglobosa*	6.56%	1.80%	NA	NA	NA	NA	NA	1.41 mg/100 g	0.17 mg/100 g	0.03 mg/100 g	(150)
6	*Dolichos lablab*	20.06–24.22%	0.33–0.75%	NA	NA	388.21–413.26 g/100 g	NA	NA	NA	NA	NA	(151)
7	*Vicia faba*	20–41%	1.2–1.9%	NA	NA	NA	NA	470 mg/100 g	NA	NA	NA	(152)

The protein content of 104 legumes across 17 families was analyzed by Prakash et al. (27). Underutilized legumes ranged in protein content from 41 to 45%. Several species of *Bauhinia* and *Canavalia gladiata* had greater protein content than soybean. In terms of amino acids, both *Bauhinia* and *Delonix* are enriched in diverse amino acids. There is a wide variety of fatty acids in the seeds of tree beans (*Parkia timoriana*) and winged beans (*Psophocarpus tetragonolobus*) (26, 28). Legumes are an excellent source of carbohydrates, fiber, and ash, but are often overlooked. *Indigofera linifolia*’s nutrient and anti-nutrient composition was reported by Siddhuraju et al. (29) and significant amounts of protein (47.2–64.2 g/kg), lipids (56.7–72 g/kg) and fiber (27.6–31.9 g/kg) were reported. Unlike *S. bispinosa*, the seeds of *I. linifolia* are packed with nutrients. Albumins and globulins are found in *I. linifolia* seeds, while globulins and glutelins are abundant in *S. bispinosa* seeds. Although both species had sufficient levels of all other essential amino acids, they were deficient in sulfur containing amino acids (25, 26). Protein makes for 20.2–293.3% of a velvet bean, while lipids account for 6.3–7.4% and carbohydrates make up 49.9–61%.

Crude lipids, calcium, magnesium, and iron are found in *Bauhinia malabarica* seeds (30). The majority was glutamic acid (45%), with just trace amounts of cystine and methionine. Seed lipids were predominantly composed of oleic acid and linoleic acid. *Vigna aconitifolia* and *V. vexillata* were analyzed (29) and found with higher protein and mineral content. The crude fat content of *V. aconitifolia* seed was higher, while the amounts of cysteine and methionine were lower. However, *V. aconitifolia* has oleic acid and palmitic acid while *V. vexillata* does not. Canavalia’s biochemical composition and nutritional value was evaluated (29) and the seeds were found to contain 31.8–36.0% protein. There is a wide range of digestible starch content (70.6–71.8%), fatty acids (71–78%) and dietary fiber (17.5–23.6%) among *Canavalia* species. Canavanine content ranged from 27% in *C. gladiata* to 42% in *C. ensiformis*. Furthermore, Ayerdi and Marraccini (18) determined that the crude protein, fat, fiber, and carbohydrate contents of *Cassia hirsuta* ranged from 15.52–21.74, 3.77–7.04, 4.68–6.92, and 62.45–70.16%, respectively. The calorie content per 100 grams of seeds was reported to vary between 1549 and 1634. The crude seed protein content of *Cassia obtusifolia* ranged from 18.52 to 22.93%, with lipids making up 5.37–7.40% and carbohydrates accounting for 57.00–60.69%. Most of the protein in the seeds was found to be globulins.

Legumes’ resistance to disease is enhanced by anti-nutrients like polyphenols, tannins, saponins, amylase inhibitors, protease inhibitors, phytic acids and lectins. Raffinose, stachyose, and verbascose can all be found in legumes. Potentially unintended consequences (31) lead to their low consumption. Legume plants contain the anti-nutrient terpenes, amongst all, saponins are sugar-containing triterpenes found in legumes such as lentils, chickpeas, soybeans, and broad beans (32). Seeds are high in proteinaceous anti-nutrients called phytohemaglutinins or lectins, which agglutinate red blood cells (33). Legumes include compounds that impede the activity of enzymes like trypsin inhibitors (34). Phenolic compounds are the most abundant class of plant defense secondary metabolites and act as anti-nutrients due to their ability to form complexes with proteins and digestive enzymes. Tannins, complex polyphenolic chemicals, reduce nutrient absorption, and are found in high concentrations in underutilized legumes. Legumes whose tannin content is high may not be as appealing as they otherwise would be. Phytic acids are present in legumes and regulate the body’s ability to absorb nutrients. They facilitate the use of proteins and are the primary source of phosphate in most seed crops. *Phaseolus lunatus* and *Phaseolus vulgaris*, respectively, have been found to contain sapogenol. Lima beans, jack beans, African yam beans, and pigeon peas are all good plant sources of the α-galactoside (35). The removal of anti-nutrients is possible through careful processing (36) that can reduce anti-nutrient components in legumes, making them safe for human consumption. It is possible to aid underutilized legumes in adapting by selecting and domesticating suitable genotypes based on anti-nutritive compounds. Thanks to advancements in biotechnology; allergenic proteins and secondary metabolites have been significantly diminished (37). Improved adaptation and long-term viability of underutilized legumes can be achieved through genome editing or marker-assisted breeding (38). Underutilized legumes are not used more widely due to a lack of knowledge, research funding, and academic interest. The existing agricultural system only allows for the cultivation of a selected few crops, yet even these have contributed to efforts to improve national and international food security. In the contemporary movement to end world hunger and improve nutrition, the use of non-traditional food sources is a fundamental tactic. The collection, preservation, and propagation of germplasms, followed by the selection of crops based on quality parameters, can improve food security.

### 2.1. Functional properties of legume-produced proteins

The behavior and performance of proteins in food systems are determined by their functionality, which includes a wide range of traits like solubility, gelation, surface activity, solubility index (%), swelling power (%), water absorption capacity (g/g), oil absorption capacity (g/g) and more. Animal proteins are widely employed in or a part of food systems because of their excellent functional qualities, which have prompted substantial scientific investigation. However, there are a number of detrimental effects associated with the creation of animal proteins. Characteristics of legume proteins are being investigated (39) in order to evaluate their potential as a source of animal protein substitution. However, such investigations have received less attention in the case of underutilized legumes. Legume hulls have significantly varying solubility indices. Black gram was found to have the maximum swelling capacity, whereas green gram was found to have the lowest. Water-holding capacity is higher in black gram and dolichos hulls, and oil-holding capacity is highest in soybean (40). The physicochemical properties of legume components are affected by the ratio of SDF (soluble dietary fiber) to IDF (insoluble dietary fiber). SDF’s hydrophilic nature gives it a WHC that is much greater than that of IDF (41). As the most abundant fraction, pectic compounds (soluble fractions) are responsible for the water-binding properties of fiber in legumes. Oil-binding capability of legume fibers is improved by the insoluble fractions (pectic polysaccharides, lignin, cellulose, and hemicellulose) (42). Due to their unique physicochemical features, DFs are able to alter the food system’s physical, rheological/textural, and sensory characteristics (43). White bread’s shelf life, rheological, physical, and sensory qualities were all enhanced by the inclusion of chickpea and soybean hulls to the formulation (44). The physicochemical characteristics of legume DFs (Dietary fibers) also have an effect on human health. The high water-holding capacity (WHC) of legume DFs, for instance, promotes transit time in the colon. By binding heavy metal ions, DFs with a high viscosity (such pectic substances) can aid in the absorption and removal of hazardous chemicals and bring down serum glucose and fat levels (45). In addition, the ratio of SDF to IDF, DF particle size, pH, environmental temperature and bile acid type; all affect the cholesterol-binding capacity of legume DFs (46) (Table 2).

**TABLE 2:** Health benefits and bioactive compounds of some underutilized legume.

SL. No.	Underutilized legumes	Bioactive compounds	Health benefits	References
1	Adzuki bean	Flavonoids: procyanidins B-1 and B-3, peonidin-3- rutinoside and malvidin-3- O-glucoside Phenolic acids: caffeic acid, ferulic acid	Antioxidant activity	(163)
		Flavonoids: catechin 7-Oβ-D-glucopyranoside (C7G), epicatechin 7-O-βD-glucopyranoside (E7G), and catechin	Antihyperglycemic activity	(164)
2	Rice bean	Phenolics: catechin, epicatechin, p-coumaric acid, ferulic acid, vitexin, isovitexin, sinapic acid, quercetin	Antioxidant activity	(165)
		Phenolics: catechin, epicatechin, p-coumaric acid, ferulic acid, vitexin, isovitexin, sinapic acid, quercetin	Antidiabetic activity	(165)
3	Horse gram	Polysaccharides: Dribose, D-arabinose, Dxylose, D-mannose, Dgalactose, and D-glucose	Antimicrobial activity	(166)
		Anthocyanins: cynidin, petunidin, delphinidin	Antioxidant activity	(167)
4	Stinky bean	Polyphenols: gallic acid, catechin, ellagic acid, quercetin	Antioxidant activity	(168)
		Phytosterols: stigmast-4- en-3-one, β-sitosterol and stigmasterol	Hypoglycemic activity	(169)

## 3. Problems related to production and climate resilient features

Orphan crops are generally more adapted to the extreme soil and climatic conditions may thrive in hot, dry climes, even when grown in rain-fed circumstances on marginal soil that exist in many parts of the world than are the major world food crops. Thus, understanding and deciphering the genetic foundation for these remarkable traits can be helpful in transferring elite characters to current growing cultivars. A plant’s morphological and physiological traits can be altered as a kind of adaptation in response to drought. Some of the ways plants react to drought include shrinking their leaves, sealing their stomata, altering the proportion of their biomass that is found in their shoots and roots, and modifying their roots (47–49). In response to drought stress, legumes’ root systems grow and are distributed in ways that optimize their ability to survive (50, 51). Root development in legumes accelerates throughout the vegetative growth stage but slows down after seed filling occurs in order to maximize the plant’s ability to take in as much soil moisture as possible (52, 53). Enhanced drought resistance in legumes has also been linked to a higher root hydraulic conductivity, which is influenced by the size and arrangement of the plant’s meta-xylem vessels (54). For instance, chickpea (*Cicer arietinum*) is a grain legume with a significantly lower root length density than barley (*Hordeum vulgare*), yet superior hydraulic conductivity allows the legume to more efficiently absorb water (55). Similarly, certain tepary bean (*Phaseolus acutifolius*) lines have deeper roots, with the biggest root mass located near the base of the soil profile. This improvement in water uptake is a direct outcome of the adaptations that have evolved. Lower stomatal conductance and smaller leaves are two further adaptive features of the bean that help it conserve water (56). Due to their hardiness and the soil microorganisms in their rhizosphere and nodules, underutilized legumes may tolerate harsh circumstances. Underutilized legumes contain unique physiological constitution and bacteria that can live and be active in harsh environments like salt, drought, pH, temperature, etc (14, 57, 58). Degefu et al. (59) found that, *Bradyrhizobium elkanii* and *Bradyrhizobium japonicum* thrive in salty and drought-stressed soils in Ethiopia and assist the pigeon pea plant. Inducing salt tolerance in mung beans with auxin and ACC deaminase-producing *Pseudomonas* and *Rhizobium* strains is also studied in details (60). Common bean was also tested for its capacity to thrive in a salty environment. Rhizobium species PvMb1, ISRA352, PvNk7, and PvNk8 alleviated salinity stress and increased plant growth and osmolyte content (glycine, betaine, and proline) (61). *Rhizobium radiobacter* from mung beans developed extracellular polymeric compounds to bioremediate arsenic and promote plant life and stress tolerance (62). Rhizobium-tolerant common beans also tolerate salt and pH. HUCRM3B, HUCRM2D, HUCRM5C, and HUCRM9C assisted common bean reduce pH and salt stress in their nodules (63). Bioremediation can be employed to put underutilized land to good use and the existence of microflora in very saline and acidic soils is evidence that these microorganisms thrive in these conditions. Pigeon pea can endure long periods of drought; thanks to its deep roots and osmotic adjustment in the leaves. Polycarpic flowering permits the crop to shed reproductive components while still maintaining photosynthetic activity, unlike drought-tolerant legumes like cowpea (64). Grass peas can withstand high water levels, low moisture levels, and moderate salt (65). The plant’s strong and extensive root system allows it to survive in nutrient-poor soil despite its fine texture, neutral to alkaline pH, and heavy clays (65). Maintaining soil fertility and reducing production costs are two benefits of grass pea’s nitrogen-fixing capabilities (65). Cowpea is a multipurpose plant grown in Africa, Asia, the Americas, and southern Europe due to its resilience in the face of adversity (66). Due to its ability to withstand acidic and alkaline soil conditions as well as its high mycorrhizal symbiosis and adequate SNF (solids not fat) levels, cowpea is tolerant to soils with low fertility (66). Cowpea may thrive in poor, sandy soils (67). In the semiarid tropics and subtropics, such as parts of Asia, Africa, Latin America and the Caribbean, pigeon pea is cultivated as a grain legume. It has a large window of maturity (90–300 days), is highly drought-resistant, and can be grown in a wide variety of climates (68). Pigeon pea’s high tolerance for acid soils and high efficiency of P uptake make it particularly exceptional (64). Future rhizofiltration systems may benefit from grass pea’s ability to retain large amounts of lead in its root tissues (69). The drought resistance of lentil, an annual legume crop used all over the world, makes it especially valuable in semiarid environments (70). The grass pea is an annual crop that serves two purposes (grain and forage) and is incredibly hardy in the face of adverse weather (71). For the rapidly growing populations in Asia and Africa, particularly in drought-prone and impoverished regions, it is one of the most promising sources of energy and protein. It’s a great option for diversifying cropping systems in Europe, Australia and the United States because it requires so few external resources (71). In “Adapting Agriculture to Climate Change,” Kew’s Millenium Seed Bank and the Global Crop Diversity Trust prioritize grass peas (72, 73). Drought-resistant grass peas are “insurance crops” for over 100 million Asian and African farmers (71). Unlike marama bean, grass pea was reported to resist drought and dehydration by modifying its maturity time, green leaf area and stomatal conductance (74, 75). Grass pea resists pests, diseases, and abiotic stress (75, 76) by producing ODAP (Oxalyldiaminopropionic acid), phenolic, flavonoid, and antioxidant compounds (72, 77) under stress conditions by scavenging the hydroxyl radicals (78).

Like soybeans and groundnuts, marama beans are high in protein and oil (79, 80) and its tubers can store water and lack nitrogen fixing nodules, thus can survive under water and nitrogen limited conditions (81). Osmotic adjustment and other drought avoidance strategies help the marama survive in extremely arid environments (82). Furthermore, plants produce many secondary metabolites and proteinaceous inhibitors to defend against environmental stresses (83). Marama bean produces a serine protease inhibitor (10.5% of its protein) which affects proteolytic activities leading to better performance in water-scarce conditions (84).

## 4. Advancements in breeding methods

The UN’s Food and Agriculture Organization (FAO) recently estimated that food and nutrition insecurity affect 800 million people, mostly in developing nations (85). One of the Sustainable Development Goals (SDG) of the 2030 Agenda, which the United Nations endorsed in September 2015 (86), was to eradicate hunger and malnutrition worldwide, with a focus on less developed nations (87). Underutilized legumes have the potential to significantly contribute to numerous SDGs by providing a highly nourishing and sustainable food supply that can aid in the fight against hunger and poverty, especially in third world nations and by promoting sustainable agriculture that is adaptable to a wide range of agroecological and production systems, requiring few, if any, synthetic fertilizers or pesticides in their cultivation. Additionally, underutilized legumes have great potential to boost food production and soil health, both of which have a positive impact on adapting to the effects of climate change and other environmental issues. Furthermore, underutilized legumes offer numerous social and economic advantages. Promoting biodiversity and incorporating underutilized crop species into peoples’ diets and food habits is a practical strategy for tackling this problem (88). Underutilized legumes have enormous genetic potential, and genetic erosion or the loss of important genetic resources is concerning. The identification and application of untapped genetic resources in the gene pools of minor crops need further study. Therefore, coordinated research efforts are required to stop the on-going loss of genetic resources among the underutilized legumes. According to recent reports, the underutilized legumes face a problem in developing efficient phenotyping and breeding methods (89). The low level of genetic diversity that breeding programmes have access to limits modern breeding efforts to increase disease resistance, quality and yield (90). Although grain legume seeds in gene banks contain a sizable amount of genetic variation, such diversity has not been completely tapped into in active breeding operations (91).

### 4.1. Conventional breeding approach

**FIGURE 1:**
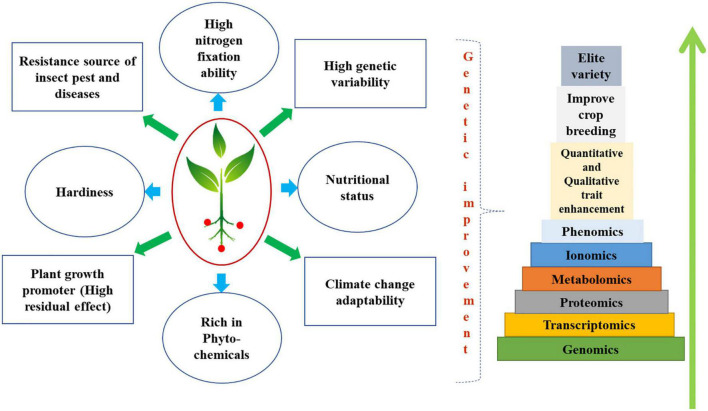
Diagram deciphering diverse omics approaches has been focused to increase nutritional compounds, reduce antinutritionals, and enhance plant qualitative as well as quantitative traits. Advances in agricultural molecular biology, crop genetics, and breeding have been made possible by the combination of conventional breeding and biotechnology. Revolutionary developments in the use of next-generation sequencing from SNP marker discovery to the whole genome-sequencing have occurred in the previous decade and these developments are expected to bring breakthroughs in crop research, particularly for underutilized crops. Molecular markers provide accurate and repeatable data on the DNA level of genetic variation across the entire genome. There has been a huge impact on crop development and the description of genetic variation thanks to these investigations of main crops. Advancements in OMICs technology have revolutionized the traditional plant breeding and emerged as one of the crucial crop-saving tool in wake of the climate change. There has been extensive use of different OMICs techniques, including Next-Generation Sequencing (NGS), transcriptomics, proteomics, and metabolomics, in the study of legumes subjected to abiotic stressors. Scientists have successfully leveraged these platforms to conduct genome-wide association analyses of linked markers known as Quantitative Trait Loci (QTL), that would enhance phytochemicals, plant growth promoters, plant nutritional status, abiotic and biotic stress tolerance, nitrogen fixation ability and wider genetic variability of underutilized legumes.

In terms of architecture, length of maturation, yield, and nutritional content, genetic and breeding efforts to improve the underutilized and neglected legume crops have not achieved the expected degree of success (92). Although traditional hybridization and other breeding methods have been attempted for some desired objectives, the expected results have not yet been obtained. On underutilized legumes, only a few successful crossings have so far been documented. On *Cajanus cajan* and certain species, there haven’t been many successes documented (93). In the African yam bean, no successful breeding lines have been reported to yet (94). Dolichos bean classification based on photoperiod sensitivity has been examined using molecular characterization utilizing SSR markers (95). Many underutilized legumes have identified reproductive obstacles, like embryo abortion, as constraints to genetic advancement. Tissue culture and micro-propagation can be utilized to produce viable haploid plants (14). Advances in DNA technology have increased our awareness of the vast potential in many plant genomes, especially underutilized legumes. Genetic engineering of cereal crops has led to genomic advancements (96). DNA-based approaches can trace plant ancestry, origin, and phylogenetic relationships (97). *Lablab purpureus, Tylosema esculentum, Vigna subterranea, V. vexillata*, and *Vigna unguiculata* are underutilized and neglected legumes (98). Diverse omics approaches have been focused to increase nutritional compounds, reduce anti-nutritionals, and enhance plant qualitative as well as quantitative traits have been deciphered in Figure 1.

### 4.2. Advanced breeding approaches - OMICS interventions

#### 4.2.1. Genomics approaches for improvement of underutilized legumes

Through the use of Next Generation Sequencing (NGS), crop genomics has undergone crucial developments during the past 10 years (99). NGS can be used to sequence the genes related to nutrients in underused crops to fulfill this criterion (100). Using data from whole-genome sequencing (WGS), it is possible to identify the genes related to nutrient accumulation, which can be subsequently used in the breeding programs (101). Previously, model legumes such as *Medicago truncatula* and *Lotus japonicus* have provided genomic information that has paved the way for advances in breeding programs and technologies like omics and genome editing to mine and transfer desirable traits for improving nutritional profile (102). These crops’ reference genomes are publicly available and help in whole genome sequencing, genotyping, and identifying genes, genomic structural variations, and SNPs (SNPs). Integrating high-throughput genotyping, phenotyping (phenomics) and crop modeling will yield useful breeding data (103). Genome sequence availability affects gene editing. The creation of a soybean reference genome was significant for agricultural legumes and legume genome research. Sanger sequencing provided eight times the WGS data, resulting in the assembly of 969.6 Mb of the 1115 Mb genome (104). Pigeonpea and chickpea draft genome sequences account for 73 and 74% of the respective genomes, respectively. Also, lentils, lupine, and mungbean genomes are being assembled. Genome sequencing results revealed stress-related genes. Genome sequencing analysis identified 187 disease-resistant chickpea genes and 111 drought-resistant pigeonpea genes (99). These genes uncovered by genome sequencing assist analyze gene candidates for substantial pressures in crop legumes. Comparing entire genome sequences offers a highly thorough understanding of the genetic relationships between different organisms. Comparative genomics plays an important role in the genome analysis of orphan crops. *Medicago truncatula* and alfalfa have nearly complete synteny between their two genomes and share highly conserved nucleotide sequences (105). The co-linearity of genes is surprisingly conserved between the two genomes, despite the pea genome being around ten times bigger than that of *Medicago truncatula* and having one fewer chromosome. The variation in chromosome number between *Medicago* and pea was suggested to be caused by chromosomal rearrangements involving *Medicago* chromosome 6 (106). A promising method called genomic selection (GS) was developed to address many of the problems with marker-assisted selection (MAS). In GS, DNA markers are utilized to predict or estimate genomic estimated breeding values (GEBV), especially for the traits complex in nature and poor heritability (99). In contrast to the large family based mapping population phenotyping and genotyping used in the MAS technique, GS can create marker-trait associations (MTAs) based on a small training population (TP) (107). GS can help in the selection of genotypes with high heritability or can eliminate genotypes that perform very poorly for the trait being selected based on GEBVs (low heritability). From the training population (TP), genotypes with higher GEBVs are chosen as parents, and crossings are performed to create candidate populations (CP). In order to fully realize the potential of GS in orphan crops robust TPs need to be generated from advanced breeding lines for which historical data on their performance has previously been acquired. Genome analysis of numerous orphan legumes has made genomic markers such as SNP readily available and thus expanding the potential for genomic selection (GS). Rychel-Bielska et al. (108) implemented the ridge regression best linear unbiased prediction (BLUP) model to predict anthracnose resistance in white lupine based on genotyping-by-sequencing (GBS)-derived SNPs. The GS model yielded an optimum predictive ability of 0.56 (108). Minor legumes have very little GS application. Thus, expanding the repertoire of genome-wide SNP markers will significantly help in implementing GS to improve future genetic gain in these legumes. Alfalfa (109), pigeonpea (110), chickpea (111), pea (112), cassava (113) and peanut are some of the other orphan crops in which GS has been employed (114). Functional genomics and plant breeding have been revolutionized by genome editing techniques, particularly the CRISPR/Cas9-based approach, which efficiently and precisely modifies specific genes of interest in plants to produce unique genetic changes (115). There are more instances of genome editing in different crops but the success rate in legume species has been relatively low (116). Soybean, cowpea, and *Medicago trancatula* are some of the legumes to which CRISPR/Cas9-mediated genome editing has been applied (117, 118). In cowpea, the CRISPR-Cas technique modifies the target gene VuSYMRK, which regulates nodule symbiosis. The result showed that the mutant plants were unable to synthesize nodules when associated with *Sinorhizobium* sp. strain NGR234 due to suppression of nodule formation (117). The detailed list of genome sequencing, protein coding genes and selected QTLs identified in underutilized legumes has been given in Table 3.

**TABLE 3:** Genome sequencing, protein coding genes and selected QTLs identified in underutilized legume.

SL. No.	Name of the species	Chromosome number	Origin	Genome size	Protein coding genes	Trait	Mapping population	QTL	References
1	Adzuki bean (*Vigna angularis var. angularis*)	2n = 2x = 22	Asiatic origin	612 mb	26,857	Seed size	*Vigna angularis* × *Vigna angularis* var. *nipponensis*	12 seed size related QTLs	(153)
2	Bambara groundnut (*Vigna subterranean*)	2n = 2x = 22	West Africa, especially Nigeria	535.05 mb	31,707	Internode length	IITA686 × Ankpa4, F2 263	One major QTL	(154, 155)
3	Cluster bean (*Cyamopsis tetragonoloba*)	2n = 2x = 14	west Africa and India	550.31 Mbp	34,680	NA	NA	NA	(156)
4	Dolichos bean (*Lablab purpureus*)	2n = 2x = 22	African origin	395.47 Mb	20,946	NA	NA	NA	(154)
5	Horsegram (*Macrotyloma uniflorum*)	2n = 20, 22	Tropical southern Asia	279.1 Mb	24,521	Drought and yield	HPK4 × HPKM249 (RIL,190)	qDFW01, qDFW02, qDTM01	(157, 158)
6	Red clover (*Trifolium pratense*)	2n = 2x = 14	European origin	418 Mbp	NA	NA	NA	NA	(159)
7	Tepary bean (*Phaseolus acutifolius*)	2n = 2x = 22	Sonoran Desert	512,626,114 bp	27,538	NA	NA	NA	(160)
8	Common vetch (*Vicia sativa*)	2n = 14	Near Eastern centre of diversity	1.8 Gb	31,146	NA	NA	NA	(161)
9	White lupin (*Lupinus albus*)	2n = 50	Mediterranean region	451 Mb	38,258	Anthracnose	Kiev × P27174 F8, RIL	antr04_1,antr05_1,antr04_2, antr05_2	(115)
10	Grass pea (*Lathyrus sativus*)	2n = 2x = 14	Central Asia and Abyssinia origin	59.7 kbp	33,819	NA	NA	NA	(162)

#### 4.2.2. Transcriptomics approaches for qualitative and quantitative improvement

The main methods for identifying potential genes involved in a biological process are transcriptomics or gene expression profiling. Twenty years ago, the only methods available for gene expression profiling were northern hybridization, serial assessments of gene expression (SAGE), microarray, etc. The situation has drastically improved over the past few years due to advancements in analytical tools and next-generation sequencing (NGSs) technologies (119). Through the sequencing of cDNA, RNA-sequencing has become a substitute for gene expression research (120). This is one of the most effective tools, widely utilized for analyzing genes that are nutrient-responsive and associated with adaptation. Transcriptome study between mungbean yellow mosaic virus (MYMV) susceptible and resistant variety revealed key genes such as JAZ and LOX genes, phytoene synthase, and cytochrome P450 that contributed to the resistance against the virus (121). Based on the transcriptome study of two distinct horse gram genotypes for drought tolerance several transcription factors (TFs) families such as WRKY, NAC and MYB were reported to be involved in conferring drought stress tolerance (122). Similar comparative transcriptome analysis of common vetch revealed various genes out of which the majority were involved in ABA oxidative stress response and cell wall modification in drought conditions (123). The development of molecular markers based on the chickpea gene that responds to drought was investigated. The development of the drought-responsive gene and molecular markers based studies on the genes in chickpea; about 435,018 reads and 21,491 ESTs were generated. Additionally, the *Medicago* genome assembly and relative genome sequencing data for chickpeas indicated 42,141 aligned tentative unique sequences (TUSs). Different markers comprised of 728 SSRs (Simple-sequence repeats), 495 SNPs (Single nucleotide polymorphisms), 387 COS (Conserved Ortholog Set), and 2088 ISRs (Inter-simple sequence repeats) were also found using the preliminary unique sequencing (124). Similar to this, the pyrosequencing method was used in another work to yield two million sequences in chickpeas, with an average length of 372 bp. The *de novo* assembly showed that the combination of long reads and short reads produced effective outcomes. With an average length of 1020 bp, about 34,760 transcripts were produced, accounting for 4.8% of the entire chickpea genome (125). About 2000 SSR markers are reported in chickpeas using transcriptome data (126). In addition, approx. 80,000 sequence tags for chickpeas were produced using whole-genome sequence profiling (127).

#### 4.2.3. Proteomics approaches for qualitative and quantitative improvement

The portion of the transcriptome that is translated into proteins is reflected in the proteome. In the field of proteome analysis, two methods are typically distinguished: a protein-based method and a peptide-based method. Proteins are isolated and quantified in the first method. The target proteins are subsequently digested, and the peptides that arise are identified using mass spectrometry. In the second method, peptide separation and quantification come before protein digestion. Since all peptide-based approaches have the drawback of losing connectivity between peptides produced from the same protein, protein-based techniques are preferred in almost all proteome investigations on orphan species (128). This guarantees that every peptide that is produced comes from the same protein. Multiple proteins are digested simultaneously in the case of protein separation methods with a lesser resolution, producing a more complicated peptide pool. The possibility of producing false positive identifications rises when a combination of masses of potentially unrelated peptides is entered into a database search. Therefore, it is essential that the parent ions undergo further Tandem mass spectrometry (MS/MS) analysis. For many years, MS/MS has been employed to gather structural data on biomolecules. The primary benefit of a protein-based approach is that protein orthologs can be successfully identified using structural data from various peptides. Furthermore, Peptide mass fingerprinting (PMF) is a method for identifying and analyzing the proteins present in a food or nutrition sample. Identifying the specific proteins, allergens or toxins, and antinutrients or anti-nutritional factors present in underutilized legumes (ULs) can be beneficial for the improvement of these legumes. When used in conjunction with the parent masses, peptide mass fingerprinting (PMF) can successfully identify peptides produced from the same protein, since MS/MS generates sequence-specific information. Peptides with a high signal-to-noise (S/Guru P. N) ratio are chosen for further fragmentation in the MS mode. In underutilized crops, such prominent peptides may occur in fewer numbers or if present, will not be informative as it is less likely that their reference will be available in the database. Due to the aforementioned reasons, the classical identification method can fail. However, when the peptide mixture only contains a small number of peptides that are all descended from a single protein, the informative peptides with a lower S/N ratio are more likely to be chosen for MS/MS analysis, increasing the likelihood of protein identification. Unfortunately, practically all software tools are created to search against a database of known proteins in an error-prone manner, which results in poor protein scores when several orphan protein peptide sequences are not similar to the annotated proteins in the database. Grass pea is one of the few orphan legumes with proteomics research (129). The seed albumin gene (AmA1) in amaranth is a non-allergenic protein that is abundant in key amino acids and largely satisfies human nutritional needs (130). Another study characterized and isolated a full-length (2076 bp long) cDNA clone from the perisperm of amaranth grain encoding a polypeptide with 606 amino acid residues, including a transit peptide of 77 amino acids. This important gene is known as the waxy gene or granule-bound starch synthase (GBSS), and it contains 1821 bp open reading frame (ORF) (131). Seed proteome analysis of *Lotus japonicus* exhibited a total of 846 unique proteins (132). Bhushan et al. (133) studied the differential expression of proteins under the moisture-stress condition in chickpeas. The study reported 134 proteins to be differentially expressed which were involved in various cellular functions, for example, cellular modifications, metabolism, and signal transduction (133). Comparative analysis of the protein profile of endosymbiotic cells produced by *Rhizobium leguminosarum* between peas and lentils revealed host-specific proteins. It signifies that the endosymbiotic bacteria rely on a combination of chemical stressors inside the nodule which are specific to the hosts (134).

#### 4.2.4. Metabolomics approaches for qualitative and quantitative improvement

Stress tolerance, inter-organismal interactions, color, taste, nutritional value, and shelf life are only few of the phenotypes that are directly impacted by the metabolites of agricultural and horticultural crops. Secondary (specialized) metabolites contribute to processes that are unique to each organism, while primary metabolites are vital for maintaining the fundamental life processes of the organism. Among the biological “omes” (i.e., genome, transcriptome, and proteome), the metabolome is thought to be the most accurate reflection of the phenotype since it includes both primary and secondary metabolites (135). Metabolite pool detection is a method for identifying the specific metabolites present in a food or nutrient sample. The detection of metabolite pools can assist in identifying the specific metabolites responsible for the distinctive flavor, aroma, and other sensory characteristics of UL. In addition, it can aid in the identification of compounds that may be toxic or detrimental to human or animal health, as well as compounds with potential health benefits, such as antioxidant or anti-inflammatory characteristics. This can inform the research and development of UL-based foods and feeds that are supplemented with these beneficial compounds and support the propagation of UL as a nutritious food source. To detect the metabolite pool of an organism, which is comprised of a wide range of chemical structures with a wide range of chemical and physical properties, the field of metabolomics primarily employs mass spectrometry (MS) and nuclear magnetic resonance (NMR) technologies, with or without chromatography (136). In the post-genomic era, several different fields of science have made use of metabolomics, which was first introduced by Nicholson et al. (137). Rathi et al. (138) identified the unique and common metabolites and their pathways responsible for the drought or dehydration response of *Lathyrus sativus* (grasspea) (138). 330 Dehydration-responsive metabolites (DRMs) were measured using chromatographic separation using high-performance liquid chromatography (HPLC) in conjunction with multiple reactions monitoring-mass spectrometry MRM-MS. The HPLC-MS data were pre-processed using mzMINE (139) and the final peak were searched against various databases such as Plant-Cyc, Kyoto Encyclopedia of Genes and Genomes (KEGG) (140), LipidMaps (9) and PubChEM (141). The metabolites belonged to 28 different functional classes. The metabolome was composed majorly of carboxylic acids (17%) followed by amino acids (13.5%). Flavonoids (10.9%), and plant growth regulators (10%) were among the compounds that constituted the metabolome. The metabolites were predominantly involved in phyto-hormone biosynthesis and osmotic adjustment. Future food security must also focus on minor crops, especially legumes in addition to the major ones (142). While the majority of orphan crops have been improved largely via conventional breeding methods, only a small number of orphan legume crops have been studied using cutting-edge technologies like genomics, transcriptomics, and metabolomics (138, 143). Omics-based breeding efforts and translational research are still lacking in many of the orphan legume crops. With the availability of high-quality genome sequences of such crops, it will be possible to apply genomic selection and prediction tools to find novel targets for selection. This will facilitate simultaneous selection for yield, disease resistance, and quality during the breeding process. It has been suggested that combining metabolome analysis with genetic analysis improves the predictability of crop attributes such as calculating lipid content and yields (144). But it has also been claimed that using metabolome data alone or in combination with other data does not improve the forecasting of production and plant height, respectively (145). This mismatch can be partially explained by the fact that environmental conditions significantly impact the plant metabolome. Further advancements in the selection of an appropriate set of metabolome data for each crop, the development of species-specific bioinformatics techniques, and the publication of an accessible dataset for bioinformatics researchers are desired in order to more accurately predict complex and environmentally dependent traits, such as yield.

## 5. Conclusion and future prospective

Throughout the review we have understood the importance of underutilized legumes in human and animal diets, role of underutilized legume’s in mitigating abiotic stress, fighting hunger, malnutrition and achieving nutritional security and the possibility of breeding stable, high yielding and resistant lines of different legumes. Moreover, the nutritional and anti-nutritional contents of these legumes help in solving the health related issues by supplying essential nutrients to humans through alternate yet necessary food sources. The major advantage of these underutilized legume’s is their ability to acclimatize to wide range of climatic conditions and evade the pest and disease problems through production of autoimmune secondary metabolites. The underutilized legumes are also rich source of proteins, vitamins, minerals, lipids, fiber and carbohydrates, which provide balanced diet in optimum quantities. They also have a major role to play in creation of employment and additional income source to rural population during periods of adverse drought and famine situations. Even though, the legumes have several agronomic and nutritional advantages over the staple cereals, but they are not gaining much popularity because of several drawbacks, like, lack of knowledge regarding these legumes among the consumers, trivial cultivation in rural areas, poor government support for production and marketing, underfunding of researches related to underutilized legume’s, over dependence on staple cereals for food security, poor accessibility to genetic materials of underutilized legume’s and non-tapping of genetic diversity of underutilized legume’s in breeding programme.

Considering the nutritional and climatic significance; the cultivation and consumption of underutilized legumes has to be encouraged by several means to achieve both food and nutritional security. The cultivation of underutilized legumes can be enhanced by provision of good quality seed material, marketing facilities and creating awareness among the consumers. Although underutilized legumes can help increase food security and lessen poverty, they aren’t always easy to sell to consumers. Not being as popular or widely consumed as other important crops like rice, wheat, or maize is a key obstacle for underutilized legumes. Because of this, marketing and selling these legumes might be challenging because they are less well-known among consumers. Some customers may be put off by the fact that underutilized legumes differ in taste, texture, and nutritional profile from more common crops. There are, nevertheless, numerous openings for the promotion and widespread adoption of underutilized legumes. Another option to boost the marketability and appeal of underutilized legumes is to process and value-add them into flour, protein powder, or other food products. Food safety and quality are other aspects that affect the marketability and customer acceptance of underutilized legumes. Growing and processing underutilized legumes using safe and sustainable practices, and then cleaning, inspecting, and packaging them properly will boost their chances of being accepted by customers. The research activities related to breeding of underutilized legumes can be funded and exhilarated. The expeditions need to be conducted throughout the world to collect germplasms of underutilized legumes and enrich their genetic diversity. The detailed study on nutritional and anti-nutritional qualities of underutilized legumes can be carried out to understand their role in human diet. Use of information on genomics, proteomics, transcriptomics and metabolomics of underutilized legume’s for breeding, pest, disease and climate resilient lines need to be encouraged. In a variety of ways, advanced breeding procedures highlighted in this review can be very beneficial for strengthening underutilized legumes. Here are a handful of such examples:

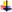

**Accelerating the breeding process:** Conventional breeding methods can be time-consuming and resource-intensive. Breeders can use advanced breeding methods such as marker-assisted selection (MAS) and genomic selection (GS) to swiftly and precisely identify desirable features. This can save time and money on breeding projects while also accelerating the development of superior legume cultivars.

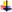

**Increasing yield and quality:** When compared to their more extensively farmed cousins, underutilized legumes frequently have lower yields and poorer quality. By discovering and selecting for specific genes associated with higher yields, disease resistance, and other desired features, advanced breeding procedures can help to increase these traits.

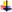

**Nutritional enhancement:** Although many underutilized legumes have a high nutritional content, they may also include anti-nutritional characteristics that limit their utilization. These characteristics can be reduced or eliminated by advanced breeding techniques, making legumes more nutritious and easier to use in food products.

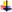

**Generating new varieties:** Modern breeding techniques can be used to develop new varieties of underutilized legumes that are better adapted to certain conditions, have higher yields or are more resistant to pests and diseases. This can help to boost crop availability and promote greater cultivation of these crops in agriculture and food systems.

Thus, in conclusion, it would rather be right to say, that legumes are the future crops that can tolerate the biotic and abiotic stresses created by anthropogenic climate change and provide the staple food for ever increasing population. Further, their inclusion in the daily diet plan can create a healthy and stable food option for both under nourished and over nourished population. Finally, the underutilized legumes can form a major subject of second green revolution, concentrating both on food security and nutritional security of the world.

## Author contributions

TB, IS, MR, PM, and DJ: conceptualization, writing – original draft preparation, and preparation of tables and supervision. SM, AKP, DK, AUP, and PG: preparation of figure and review and editing. All authors read and approved the manuscript.
